# Genome-Wide Identification of Genes Important for Growth of *Dickeya dadantii* and *Dickeya dianthicola* in Potato (*Solanum tuberosum*) Tubers

**DOI:** 10.3389/fmicb.2022.778927

**Published:** 2022-01-25

**Authors:** Tyler C. Helmann, Melanie J. Filiatrault, Paul V. Stodghill

**Affiliations:** ^1^Emerging Pests and Pathogens Research Unit, Agricultural Research Service, United States Department of Agriculture, Robert W. Holley Center for Agriculture and Health, Ithaca, NY, United States; ^2^Plant Pathology and Plant-Microbe Biology Section, School of Integrative Plant Science, Cornell University, Ithaca, NY, United States

**Keywords:** potato, soft rot, RB-TnSeq, TnSeq, *Dickeya dadantii*, *Dickeya dianthicola*

## Abstract

*Dickeya* species are causal agents of soft rot diseases in many economically important crops, including soft rot disease of potato (*Solanum tuberosum*). Using random barcode transposon-site sequencing (RB-TnSeq), we generated genome-wide mutant fitness profiles of *Dickeya dadantii* 3937, *Dickeya dianthicola* ME23, and *Dickeya dianthicola* 67-19 isolates collected after passage through several *in vitro* and *in vivo* conditions. Though all three strains are pathogenic on potato, *D. dadantii* 3937 is a well-characterized model while *D. dianthicola* strains ME23 and 67-19 are recent isolates. Strain ME23 specifically was identified as a representative strain from a 2014 outbreak on potato. This study generated comparable gene fitness measurements across ecologically relevant conditions for both model and non-model strains. Tubers from the potato cultivars “Atlantic,” “Dark Red Norland,” and “Upstate Abundance” provided highly similar conditions for bacterial growth. Using the homolog detection software PyParanoid, we matched fitness values for orthologous genes in the three bacterial strains. Direct comparison of fitness among the strains highlighted shared and variable traits important for growth. Bacterial growth in minimal medium required many metabolic traits that were also essential for competitive growth *in planta*, such as amino acid, carbohydrate, and nucleotide biosynthesis. Growth in tubers specifically required the pectin degradation gene *kduD*. Disruption in three putative DNA-binding proteins had strain-specific effects on competitive fitness in tubers. Though the Soft Rot *Pectobacteriaceae* can cause disease with little host specificity, it remains to be seen the extent to which strain-level variation impacts virulence.

## Introduction

The Soft Rot *Pectobacteriaceae* comprise *Dickeya* and *Pectobacterium* species that are the causal agents of bacterial soft rot diseases on economically important vegetables and ornamentals (Adeolu et al., 2016; Motyka et al., 2017). These necrotrophic pathogens rely on numerous traits to survive the various stress conditions encountered in the host, including oxidative stress, osmotic stress, iron starvation, and toxic compounds (Jiang et al., 2016; Reverchon et al., 2016). During initial plant colonization, cells encounter a slightly acidic apoplast (pH 5.0–6.5) or tuber (pH 5.5–6.2), and pH levels increase to slightly basic (pH 8) by the late stage of infection (Grignon and Sentenac, 1991; Nachin and Barras, 2000; Kiszonas and Bamberg, 2010). At high cell densities, the production of plant cell wall degrading enzymes is induced, leading to the maceration of plant tissues and the formation of visible symptoms (Reverchon et al., 2016). In response to these changing conditions, cells must respond to environmental cues to adapt global expression of stress response genes and virulence factors (Jiang et al., 2016; Raoul des Essarts et al., 2019).

The taxonomy of the *Dickeya* genus has undergone substantial revision with the addition of novel species *D. solani*, *D. aquatica*, and *D. fangzhongdai* (Samson et al., 2005; Parkinson et al., 2014; Van Der Wolf et al., 2014; Tian et al., 2016). However, an increase in available whole-genome sequence data has improved species-level identification based on pairwise average nucleotide identity (ANI), *in silico* DNA-DNA hybridization (*is*DDH), and core genome multilocus sequence analysis (MLSA) (Zhang et al., 2016). There is little known about host-specific traits, as these species generally have broad host ranges (Van Gijsegem et al., 2021). In addition, there are no known resistance genes for potato soft rot, and it is therefore impossible to predict cultivar resistance without testing (Lyon, 1989; Czajkowski et al., 2011; Chung et al., 2013). Without gene-for-gene resistance, potato cultivar tolerance is reliant on physical barriers and antimicrobial small molecules such as phenolics or the phytoalexin rishitin (Lyon, 1989). An alternative strategy being explored is the use of bacteriophage-based biocontrol for potato plants and tubers, particularly of the highly virulent *D. solani* (Adriaenssens et al., 2012; Czajkowski et al., 2017). *Dickeya* virulence factors and transcriptional regulators of virulence genes are generally conserved. Studies in *D. solani* have suggested a closed pangenome with many conserved virulence factors and transcriptional regulators (Golanowska et al., 2018; Motyka-Pomagruk et al., 2020). However, virulence regulon differences indicate some virulence genes could have differential expression among strains (Golanowska et al., 2018). Pangenomic analysis of *D. dianthicola* also reflects a closed pangenome, though almost all sequenced strains were originally isolated from potato (Ge et al., 2021c).

Transposon mutagenesis followed by high-throughput sequencing (TnSeq) is a valuable screening tool to identify genes important for growth in a given condition. Gene fitness is functionally defined as the change in relative mutant abundance within a population and is a quantitative measure of growth rate (van Opijnen et al., 2009). TnSeq has been used to identify *D. dadantii* genes important for growth in chicory (Royet et al., 2019). This work identified several metabolic pathways essential for *in planta* growth, primarily those involved in biosynthesis of nucleotides, amino acids, and some vitamins (Royet et al., 2019). A modification of TnSeq to add 20-nucleotide DNA “barcodes” to transposon donor plasmids, known as random barcode transposon-site sequencing (RB-TnSeq) enables highly scalable TnSeq assays (Wetmore et al., 2015). This method has been applied to over 44 bacterial strains to date (Price et al., 2018), including plant pathogenic *Pseudomonas* spp. and *Ralstonia* spp. (Cole et al., 2017; Helmann et al., 2019; Georgoulis et al., 2021). A motivating factor for this study was to demonstrate the value of RB-TnSeq to characterize necrotrophic plant pathogenic bacteria *in planta*; using both an established model strain as well as recently isolated and uncharacterized strains (Liu and Deutschbauer, 2018).

To identify bacterial traits important for growth in potato (*Solanum tuberosum*) tubers, we examined three strains across two species: *D. dadantii* 3937 (*Dda*3937), *D. dianthicola* ME23 (*Ddia*ME23), and *D. dianthicola* 67-19 (*Ddia*6719). While these three strains are all pathogenic on potato, *Dda*3937 was originally isolated from *Saintpaulia ionantha* (Lemattre and Narcy, 1972), and *Ddia*6719 was recently isolated from New Guinea impatiens (*Impatiens hawkeri*) (Liu et al., 2020a,b). *Dda*3937 has been a model strain used for molecular studies since its isolation in 1972 (Lemattre and Narcy, 1972), while *Ddia*ME23 was isolated as a representative strain for a 2014 potato disease outbreak (Ma et al., 2019). Pairwise ANI between *Dda*3937 and *Ddia*ME23 is 92.8% (Chen et al., 2019). Using barcoded transposon insertion mutant libraries in these three strains, we screened for genes that contributed to competitive fitness during growth *in vitro* and in potato tubers. By leveraging RB-TnSeq in a shared susceptible host for *D. dadantii* and *D. dianthicola*, we aimed to identify common and unique virulence factors among representative strains for these two species.

## Materials and Methods

### PyParanoid Gene Ortholog Group Assignments

Gene ortholog groups were generated using the PyParanoid analysis pipeline v0.4.1 (Melnyk et al., 2019). Peptide sequences from the following RefSeq genome assemblies were used to construct ortholog groups: GCF_000147055.1 (*Dda*3937), GCF_003403135.1 (*Ddia*ME23), and GCF_014893095.1 (*Ddia*6719). From these assemblies, RefSeq gene loci were then matched to their corresponding protein names to allow comparison to the Barcode Sequencing (BarSeq) fitness data. Additionally, Clusters of Orthologous Groups (COG) categories for *Dda*3937 were downloaded from the IMG database (Chen et al., 2019), GenBank gene names were replaced with their corresponding RefSeq names, and added to this ortholog table, allowing putative COG assignments for orthologous genes in *D. dianthicola* strains.

### Barcoded Transposon Library Construction

Strains used in this study are described in Supplementary Table 1. All bacteria were cultured in Luria-Bertani (LB) medium (10 g tryptone, 5 g yeast extract, and 10 g NaCl per 1 L) (Bertani, 1951) at 28°C, except for pure culture *E. coli* grown at 37°C. When noted, kanamycin was used at a final concentration of 50 μg/ml. Barcoded transposon libraries were constructed by conjugating the barcoded *mariner* transposon plasmid pKMW3 from the *E. coli* WM3064 donor library APA752 (Wetmore et al., 2015) into wild-type *Dda*3937, *Dia*ME23, and *Ddia*6719. Recipient *Dickeya* strains were grown as 3 ml LB liquid cultures overnight and 1.5 ml of each culture was subcultured into 30 ml LB without antibiotics. An entire 1 ml freezer aliquot of the *E. coli* donor library was thawed and used to inoculate 25 ml LB containing 300 μM diaminopimelic acid (DAP) (Sigma-Aldrich, United States) and kanamycin. All cultures were grown to OD_600_ of approximately 1.0, washed with 10 mM KPO_4_, and combined in equal amounts before plating donor-recipient pairs each on 50 LB plates containing 300 μM DAP. Conjugations were incubated at 28°C overnight, and exconjugants were then scraped into 40 ml 10 mM KPO_4_. This conjugation mixture was then vortexed, spread onto 200 LB kanamycin plates per strain, and incubated at 28°C for 3 days. All colonies were resuspended in 200 ml LB with kanamycin, diluted back to a starting OD_600_ of 0.2, and grown at 28°C with shaking for 6–8 h, to a concentration of approximately 10^9^ CFU/ml (measured as a 1/10 dilution at OD_600_ = 0.15–0.3). Glycerol was added to the library to a final concentration of 15%, and 1 ml aliquots were frozen at –80°C.

### DNA Library Preparation and Sequencing

For DNA library preparation, genomic DNA from each library was purified from an entire 1 ml cell pellet using the Monarch Genomic DNA Purification Kit (New England Biolabs, United States). Samples were eluted in 50 μl nuclease-free water. Purified DNA was quantified on a NanoDrop One (Thermo Fischer Scientific, United States), and 500 ng DNA was used as input for the NEBNext Ultra II FS DNA Library Prep kit (New England Biolabs, United States), following the manufacturer’s instructions with modifications as follows. For enzymatic DNA fragmentation, a 12-min incubation time was used. DNA fragments were size selected using AMPure XP magnetic beads (Beckman Coulter, United States) at the recommended ratios 0.4X and 0.2X. We used a modified version of the protocol described in Wetmore et al. (2015), with a two-step PCR used to enrich for transposon insertion sites, based on (Rubin et al., 2021). A custom splinkerette adapter was ligated to fragmented DNA, prepared by annealing oligos:/5Phos/G*ATCGGAAGAGCACACGTCTGGGTTTTTT TTTTCAAAAAAA*A and G*AGATCGGTCTCGGCATTCCC AGACGTGTGCTCTTCCGATC*T (Rubin et al., 2021). Between rounds of PCR and before submitting for sequencing, DNA was cleaned by binding to AMPure XP magnetic beads, using a bead ratio of 0.9X and eluted in 15 μl 0.1X TE buffer for intermediate steps and 30 μl 0.1X TE for sequencing. Finally, the sequencing library was quantified using a Qubit dsDNA HS assay kit (Thermo Fischer Scientific, United States). DNA libraries were submitted for sequencing at the Biotechnology Resource Center (BRC) Genomics Facility at the Cornell Institute of Biotechnology on an Illumina MiSeq to check library quality, followed by sequencing on a NextSeq 500 (Illumina, Inc., United States). All mapping used single-end sequencing for 150 bp fragments.

### Transposon Library Mapping

Sequence data were analyzed using the scripts from the FEBA package v1.3.1 (Wetmore et al., 2015). MapTnSeq.pl was used to identify the barcode and location in the genome of each read with identifiable transposon sequence from both MiSeq and NextSeq reads, based on the “model_pKMW3.2” sequence. DesignRandomPool.pl was used to assemble the mutant pool using barcodes seen in a single location 10 or more times. All TnSeq mapping and BarSeq fitness calculation code is available at http://bitbucket.org/berkeleylab/feba/ (Wetmore et al., 2015). Mapping scripts were run on a Cornell University BioHPC Cloud 40-core Linux (CentOS 7.6). server with 256 GB RAM.

### Gene Essentiality Predictions

Using the output from MapTnSeq.pl, gene essentiality predictions were made using https://bitbucket.org/berkeleylab/feba/src/master/bin/Essentiality.pl and the function “Essentials” from https://bitbucket.org/berkeleylab/feba/src/master/lib/comb.R (Wetmore et al., 2015). Using the median insertion density and the median length of genes > 100 bp, this method calculates how short a gene can be and still be unlikely to have no insertions by chance (*P* < 0.02, Poisson distribution); genes shorter than this threshold are then excluded (Price et al., 2018). For the *Dickeya* strains examined here, the minimum gene length for a gene to be predicted as essential for growth in LB was 175 bp (*Dda*3937) or 150 bp (*Ddia*ME23 and *Ddia*6719). Protein-coding genes are then considered to be essential or nearly essential if there are no fitness values and the normalized central insertion density score and normalized read density score as computed by the FEBA package were < 0.2 (Price et al., 2018).

### Library Pre-culture

For a given BarSeq experiment, a single transposon library freezer aliquot was thawed and recovered in 25 ml LB containing kanamycin at 28°C until OD_600_ = 0.5–0.7, approximately 6–8 h. At this point, two 1 ml cell pellets were frozen as time0 measurements, and the remaining culture was washed in 10 mM KPO_4_ and used to inoculate experimental samples.

### *In vitro* Samples

All *in vitro* cultures were grown in 1 ml volumes in 24-well plates. In each well, 50 μl starter culture at 0.3 OD_600_ was added to 950 μl medium containing kanamycin. Media tested were LB, Potato Dextrose Broth (PDB)(pH 5) (Sigma-Aldrich, United States), and M9 minimal medium (M9) as described in M9 Minimal Medium (Standard) (2010) but containing 0.4% glycerol instead of 0.4% glucose. Plates were incubated at 28°C with shaking at 200 rpm. After 1 day (LB and PDB) or 2 days (M9), each 1 ml sample was pelleted and frozen prior to genomic DNA extraction.

### Tuber Samples

Prior to inoculation, all tubers were rinsed and then surface sterilized by submerging in 70% ethanol for 10 min, followed by two washes with distilled water. Inoculum was standardized to 10^9^ CFU/ml by measuring a 1/10 culture dilution at OD_600_ = 0.3 (corresponding to 10^8^ CFU/ml), and 10 μl was inoculated in two replicate stab wounds created by pushing a 200 μl pipet tip roughly 3 mm into each tuber. Six replicate tubers were used for each bacterial strain and potato cultivar. Inoculated tubers were stored in plastic bags at 28°C. Two days after inoculation, ∼2 cm length cores were taken at each site of inoculation using a 1 cm diameter cork borer. Duplicate cores from each tuber were pooled in 8 ml 10 mM KPO_4_ and shaken at 200 rpm at 28°C for 10 min. For each sample, 2 ml of bacterial suspension were pelleted and frozen prior to DNA extraction.

### BarSeq PCR and Sequencing

Genomic DNA was purified from cell pellets using the Monarch Genomic DNA Purification Kit (New England Biolabs, United States). Purified DNA samples were eluted in 30 μl nuclease-free water and quantified on a Nanodrop One (Thermo Fischer Scientific, United States). After gDNA extraction, the 98°C BarSeq PCR as described in Wetmore et al. (2015) was used to specifically amplify the barcode region of each sample. The PCR for each sample was performed in 50 μl total volume: containing 0.5 μl Q5 High-Fidelity DNA polymerase (New England Biolabs, United States), 10 μl 5X Q5 buffer, 10 μl 5X GC enhancer, 1 μl 10 mM dNTPs, 150–200 ng template gDNA, 2.5 μl common reverse primer (BarSeq_P1), and 2.5 μl of forward primer from one of the 96 indexed forward primers (BarSeq_P2_ITXXX), both at 10 μM (Wetmore et al., 2015). Following the BarSeq PCR, 10 μl of each reaction was pooled (46–49 samples per pool), and 200 μl of this DNA pool was subsampled and purified using the DNA Clean and Concentrator Kit (Zymo Research, United States). The final DNA sequencing library was eluted in 30 μl nuclease-free water, quantified on a Nanodrop One, and submitted for sequencing at the BRC Genomics Facility at the Cornell Institute of Biotechnology. Prior to sequencing, the quality of each amplicon pool was assessed using a Bioanalyzer. Each sequencing pool was run on a single NextSeq 500 (Illumina, Inc., United States) lane for 75 bp single-end reads.

### Gene Fitness Calculations

Sequencing reads were used to calculate genome-wide gene fitness using the FEBA scripts MultiCodes.pl, combineBarSeq.pl, and BarSeqR.pl (Wetmore et al., 2015). Scripts to calculate gene fitness values were run on a Cornell University BioHPC Cloud 40-core Linux (CentOS 7.6). Server with 256 GB RAM. From the raw fastq reads for each sample, individual barcode sequences were identified and counted using MultiCodes.pl, and these counts were combined across samples for a given transposon library using combineBarSeq.pl. Using BarSeqR.pl, fitness values for each insertion strain were calculated as the log_2_ ratio of barcode abundance following library growth under a given experimental condition divided by the abundance in the time0 sample. The fitness of each gene is the weighted average of strain fitness values based on the “central” transposon insertions only, i.e., those within the central 10–90% of a gene. Barcode counts were summed between replicate time0 samples. For analysis, genes were required to have at least 30 reads per gene in the time0 sample, and 3 reads per individual strain (Wetmore et al., 2015). Gene fitness values were normalized across the chromosome so that the median gene fitness value was 0. All experiments described here passed previously described quality control metrics (Wetmore et al., 2015; Price et al., 2018).

### Fitness Analysis and Plotting

We focused on genes having fitness values > 1 or < –1 and absolute t-like test statistic > 4. This t score is an estimate of the reliability of the fitness measurement for a gene, and is equal to the fitness value divided by the square root of the maximum variance calculated in two ways (Wetmore et al., 2015). With these cutoffs, we also calculated gene fitness values comparing replicate time0 samples (Price et al., 2018; Liu et al., 2021). Across 6 (each *Dda*3937 and *Ddia*ME23) and 2 (*Ddia*6719) replicate time0 samples, 0 gene fitness values had fitness > 1 or < –1 and absolute t > 4. Data were analyzed in R v4.0.3 (R Core Team, 2017) and visualized using the package ggplot2 v3.3.5 (Wickham, 2016). The principal components analysis was performed on the gene fitness matrix for each *Dickeya* strain using the R function prcomp, which performs centered singular value decomposition.

### Data Availability Statement

All raw Illumina reads used for mapping and fitness assays have been deposited in the Sequence Read Archive under BioProject accession number PRJNA692477. Individual sample accession numbers are listed in Supplementary Table 2. Annotated scripts used for computational analysis are available at http://github.com/tylerhelmann/dickeya-barseq-2021. Experimental fitness values are publicly available on the interactive Fitness Browser at http://fit.genomics.lbl.gov.

## Results

### Identification of Homologous Gene Families in *Dickeya dadantii* 3937, *Dickeya dianthicola* ME23 and *Dickeya dianthicola* 67-19

To enable direct comparison of gene fitness measurements between strains, we constructed a database of homologous gene families using the PyParanoid analysis pipeline (Melnyk et al., 2019). Based on clustering of all predicted protein sequences from *Dda*3937, *Ddia*ME23, and *Ddia*6719, 3,821 total homolog groups were identified, representing 88.1% of the total input sequences. Of these, 3,310 groups contained single-copy genes in all three strains. For each group, gene loci, protein identifiers, and gene descriptions are listed in Supplementary Table 3. This table also contains COG assignments matched from the *Dda*3937 IMG genomic annotation (Chen et al., 2019).

### Creation of Barcoded Transposon Libraries in *Dickeya* spp.

To measure contributions of individual genes to fitness, we constructed barcoded transposon mutant libraries in the *Dickeya* strains using a barcoded *mariner E. coli* donor library (Wetmore et al., 2015). These libraries ranged in size from 334,893 to 541,278 mapped genomic insertional strains, with 37–62 median strains per hit gene (Table 1). Of the three strains tested, only one gene in the *Ddia*6719 genome did not contain any TA dinucleotide sites and was therefore inaccessible to the *mariner* transposon. Mapped insertions were evenly distributed across the chromosome of each strain (Supplementary Figure 1).

**TABLE 1:** Characteristics of the *mariner* transposon libraries.

Library	Insertions in genome	Central insertion strains	Genes with central insertions (Total)	Median strains per hit gene
*D. dadantii* 3937	337,541	193,696	3,882 (4,213)	37
*D. dianthicola* ME23	541,278	321,087	3,805 (4,182)	62
*D. dianthicola* 67-19	334,893	200,170	3,728 (4,110)	41

*“Central” insertions are those within the central 10–90% of a gene.*

### Identification of Essential Gene Sets in *Dickeya dadantii* and *Dickeya dianthicola*

Based on analysis of the TnSeq mapping data, essential genes were predicted using the FEBA RB-TnSeq analysis pipeline (Wetmore et al., 2015; Price et al., 2018). We identified 374–426 genes per strain that are likely to encode essential or near-essential genes for growth in LB (Supplementary Table 4). Using the ortholog group assignments for these genes, 316 of these predicted essential genes (74–84%) are shared among all three strains (Supplementary Figure 2). Most predicted essential genes are in the functional categories of “Translation, ribosomal structure, and biogenesis,” “Cell wall/membrane/envelope biogenesis,” “Coenzyme transport and metabolism,” “Energy production and conversions,” and “Replication, recombination, and repair” (Supplementary Table 5).

### Conducting Pooled Growth Assays to Measure Relative Mutant Fitness

To generate genome-wide gene fitness values for the barcoded transposon libraries, each strain was grown in the rich media LB and Potato Dextrose Broth (PDB) as well as M9 minimal medium supplemented with 0.4% glycerol (Supplementary Figure 3). Strain fitness values were calculated as a log2 ratio of barcode abundance following sample growth with barcode abundance measured in the time0 duplicate samples. Gene fitness is the weighted average of individual strain fitness values (Wetmore et al., 2015). For fitness calculations, insertions in the first and last 10% of coding regions were excluded, with insertions in the remaining 80% of the gene considered “central.” While 91–92% of genes in all strains contained centrally mapped insertions, not all genes were used in fitness calculations due to low read or insertion abundance. We focused our analysis on genes with fitness values > 1 or < –1, and absolute t-score > 4 (Supplementary Table 6). Across all conditions, we calculated fitness values for 3,705 (*Dda*3937), 3,761 (*Ddia*ME23), and 3,528 (*Ddia*6719) genes, representing 88, 90, and 86% of the total genes in each strain, respectively.

**FIGURE 1:**
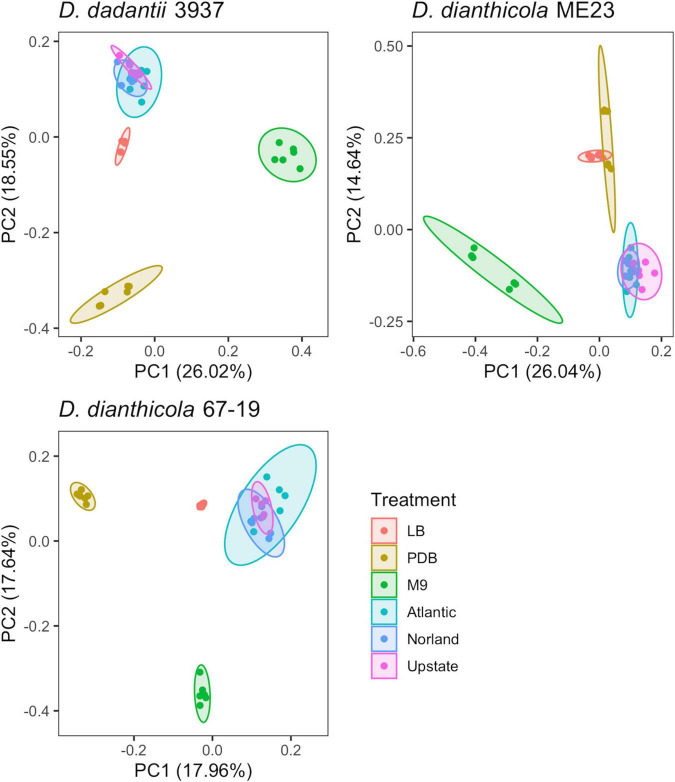
PCA of experimental samples based on fitness values calculated for *D. dadantii* 3937, *D. dianthicola* ME23, and *D. dianthicola* 67–19. Available fitness values for each sample, respectively: *N* = 3,705; 3,761; 3,528. Superimposed ellipses are based on a multivariate t-distribution.

Principal component analysis showed gene fitness values of the three tuber conditions overlapped (Figure 1), and so these samples were jointly considered as a single “Tuber” condition for some subsequent analyses.

### Disruption Mutants With Fitness Defects in Rich Media

**FIGURE 2:**
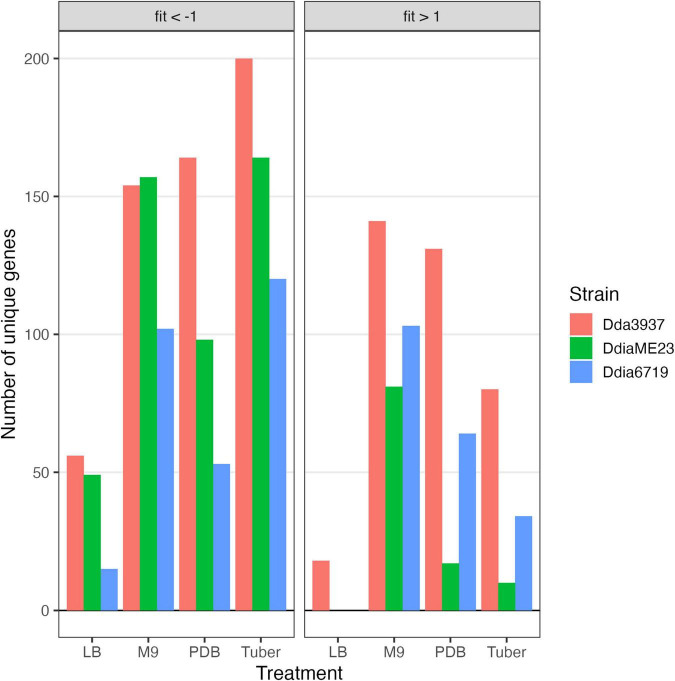
Number of unique genes for each condition with fitness values of < –1 or > 1, and absolute t-like test statistic > 4 in at least one replicate sample.

**FIGURE 3:**
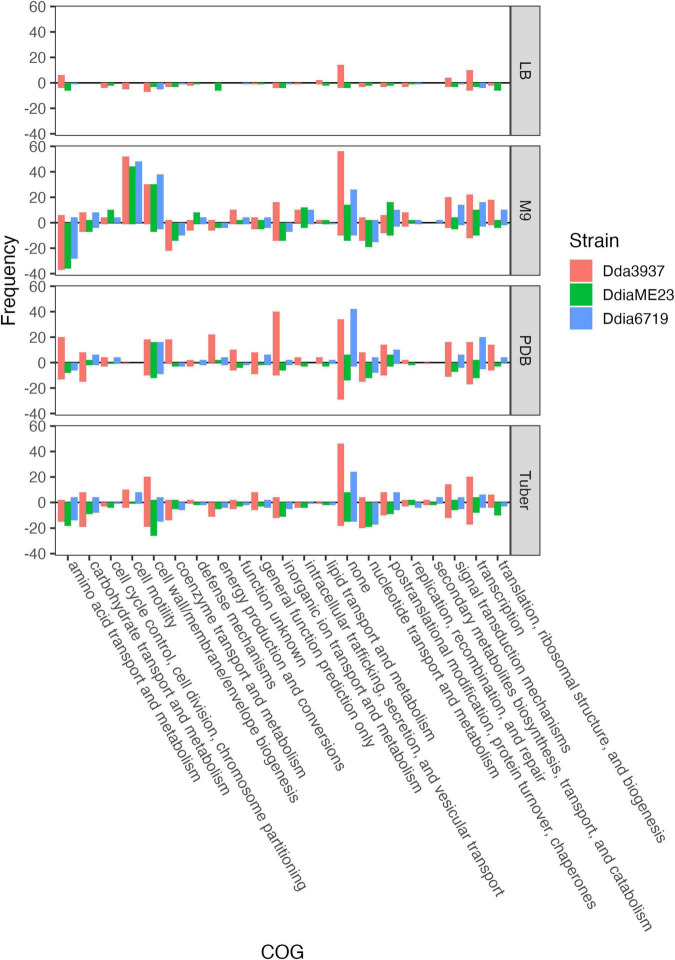
Number of unique genes for each condition with fitness values of < –1 or > 1, and absolute t-like test statistic > 4 in at least one replicate sample. Genes where fit < –1 are show below the line *y* = 0, while genes where fit > 1 are shown above. COG assignments are based on the *D. dadantii* 3937 annotation in the IMG database (Chen et al., 2019), and extrapolated to *D. dianthicola* ME23 and *D. dianthicola* 67-19 based on PyParanoid-generated ortholog groups.

As the libraries were constructed on LB medium, relatively few mutations deleterious in LB were maintained in the populations (Figure 2). Figure 3 presents these data split by COG category. Limited mutations in genes categorized as “cell wall/membrane/envelope biogenesis” (*mdoGH*) and “cell cycle control, cell division, chromosome partitioning” (*ftsX*) were present in the mapped populations but generally detrimental in LB for all three strains (Supplementary Figure 4). Even in LB, some variation was apparent between strains, such as disruptions in the gene encoding the cell division protein ZapB which decreased competitive fitness in *Dda*3937 but not *Ddia*ME23 or *Ddia*6719 (Supplementary Figure 4).

The rich medium PDB provided a very different gene fitness profile than LB, likely due to nutritional differences and its slightly acidic nature (pH 5). For all three strains, more genes were detrimental (fit > 1) and beneficial (fit < −1) in PDB relative to LB (Figure 2). Genes in diverse metabolic categories contributed to competitive fitness, including “amino acid transport and metabolism,” “carbohydrate transport and metabolism,” “cell wall/membrane/envelope biogenesis,” “coenzyme transport and metabolism,” “inorganic ion transport and metabolism,” “nucleotide transport and metabolism,” “signal transduction mechanisms,” “transcription,” and “translation, ribosomal structure, and biogenesis” (Figure 3). For example, in all three strains oligopeptidase A and the low affinity potassium transporter Kup were specifically important in PDB for growth (Supplementary Figure 5). Disruptions in the two-component system RtsAB were specifically beneficial for *Dda*3937 in PDB, as were disruptions in the zinc uptake transcriptional repressor Zur (Supplementary Figure 5). Though LB and PDB are both complex rich media, pH and/or specific available nutrients differed enough to clearly separate the gene fitness profiles for *Dda*3937 and *Ddia*6719, though not for *Ddia*ME23 (Figure 1).

### Disruption Mutants With Fitness Defects in Minimal Medium

**FIGURE 4:**
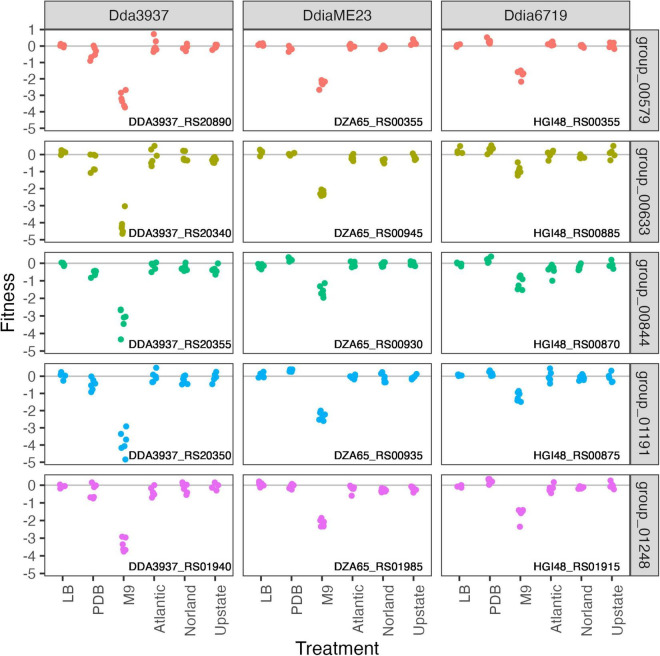
Mutations in arginine biosynthetic genes are detrimental only in M9 minimal medium. Gene fitness values for argininosuccinate synthase ArgG (group 00579), argininosuccinate lyase ArgH (group 00633), acetylornithine deacetylase ArgE (group 00844), N-acetyl-gamma-glutamyl-phosphate reductase ArgC (group 01191), and ornithine carbamoyltransferase ArgF (group 01248).

**FIGURE 5:**
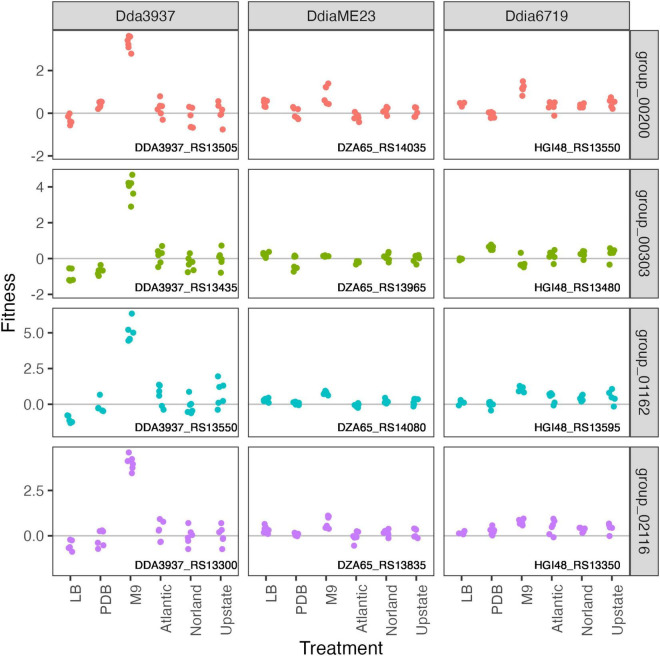
Mutations in flagellar-associated genes increase competitive fitness of *D. dadantii* 3937 in M9 minimal medium. Gene fitness values for flagellar biosynthesis protein FlhA (group 00200), flagellar hook-associated protein FlgK (group 00303), flagellar motor protein MotB (group 001162), and RNA polymerase sigma factor FliA (group 02116).

In the minimal medium M9 containing glycerol as a carbon source, important genes included categories such as “amino acid transport and metabolism,” “carbohydrate transport and metabolism,” “coenzyme transport and metabolism,” and “nucleotide transport and metabolism.” While many amino acids were limiting in both M9 and tuber samples, arginine biosynthetic genes (*argCEFGH*) were uniquely important in M9, suggesting the presence of available arginine in tubers (Figure 4). Conversely, mutations in many “cell motility” genes had a large positive effect, though this effect was often limited to *Dda*3937 (Figure 5). This is indicative of the limited energy available in minimal medium, and the high energy cost of motility.

### Genes Contributing to Growth in Tubers

**FIGURE 6:**
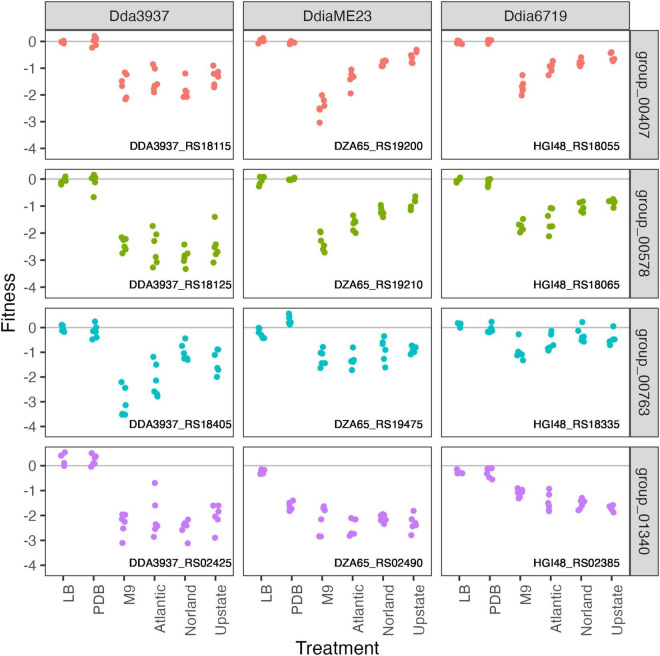
Amino acid biosynthetic genes important in M9 minimal medium as well as growth in potato tubers. Gene fitness values for 2-isopropylmalate synthase LeuA (group 00407), 3-isopropylmalate dehydratase large subunit LeuC (group 00578), threonine synthase ThrC (group 00763), and phosphoserine phosphatase SerB (group 01340).

To calculate genome-wide gene fitness values in an ecologically and economically relevant condition, we inoculated the transposon libraries into tubers of three potato cultivars: “Atlantic,” “Dark Red Norland,” and “Upstate Abundance.” As each transposon library contains over 300,000 unique strains, we inoculated approximately 10^7^ cells into each tuber (10 μl of a 10^9^ CFU/ml solution). After 2 days incubation at high humidity, we recovered cells by streaming for barcode sequencing and calculation of gene fitness values. Many genes involved in amino acid biosynthesis that were important for growth in M9 were also important in tubers (*leuAC, thrC, serB*), highlighting potentially limiting factors for growth during potato soft rot (Figure 6).

**FIGURE 7:**
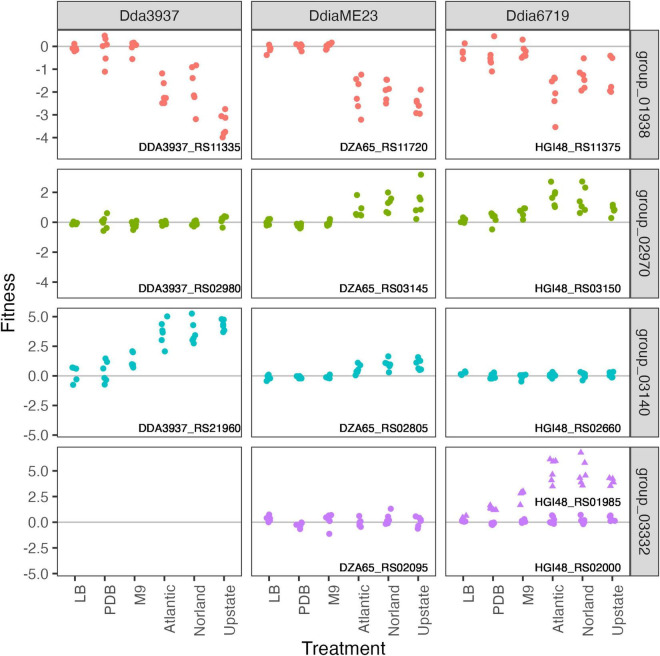
In tubers, pectin degradation contributes to growth, while the role of putative DNA-binding proteins varies among strains. Pectin degradation by 2-dehydro-3-deoxy-D-gluconate 5-dehydrogenase KduD (group 01938) is specifically important for growth in tubers. Gene fitness values shown for three putative DNA binding proteins: ortholog groups 02970, 03140, and 03332. No ortholog was detected in *D. dadantii* 3937 for group 03332 (helix-turn-helix transcriptional regulators). In *D. dianthicola* 67-19, there are two genes in group 03332 (HGI48_RS01985, triangles; HGI48_RS02000, circles). All other orthogroup members are single-copy in each strain.

The pectin degradation protein 2-dehydro-3-deoxy-D-gluconate 5-dehydrogenase KduD was specifically important for growth in tubers (Figure 7). Interestingly, we identified several putative DNA-binding or helix-turn-helix transcriptional regulators where mutant strains had strain-specific increased fitness in tubers (Figure 7). Insertions in the *Ddia*6719 helix-turn-helix transcriptional regulator *HGI48_RS01985* increased fitness in tubers, while insertions in the paralog *HGI48_RS02000* had no effect on fitness. There is no ortholog for this gene in *Dda3937*, and the ortholog in *Ddia*ME23 had no disruption phenotype in any condition tested (Figure 7).

## Discussion

The soft rot infection process is complex, requiring tolerance of stress conditions and coordination of disease-stage specific virulence traits (Jiang et al., 2016; Reverchon et al., 2016). Despite taxonomic and diagnostic improvements within the soft rot *Pectobacteriaceae*, our molecular understanding of these pathogens remains highly reliant on the model strain *D. dadantii* (previously *Erwinia chrysanthemi*) 3937 (Toth et al., 2011; Van Gijsegem et al., 2021). However, *D. dianthicola* strains have been among those more recently responsible for outbreaks in the United States and globally (Toth et al., 2011; van der Wolf et al., 2021). It is therefore important to consider common and variable virulence traits of *Dickeya* species; to better understand the infection processes and associated risk factors.

In this study, we focused specifically on bacterial colonization of potato tubers, an ecologically important infection model. *Dickeya* species share many common virulence strategies, including the production of plant cell wall degrading enzymes such as pectate lyases (Reverchon et al., 2016). However, virulence traits can vary even among closely related strains. For example, Ge et al. (2021b,c) reported the *D. dianthicola* strains isolated in the United States comprise multiple genetic groups, with differing type IV and type VI secretion system genes. These differences, determined by pan-genomic analysis, suggest driving forces for population level changes in outbreaks which can be explored by molecular characterization (Ge et al., 2021c). By constructing barcoded transposon insertion libraries in *D. dadantii* and *D. dianthicola*, and testing these mutant populations in parallel conditions, we were able to directly compare gene contributions to fitness among these related strains. The scalability of RB-TnSeq, paired with ortholog prediction or gene co-fitness measurements, has proven to be a useful method to compare ortholog presence and function (Price et al., 2018). By measuring genome-wide gene contributions to growth, we identified a comprehensive list of genes in three *Dickeya* strains that contribute to fitness in three *in vitro* conditions and tubers. This approach could be applied to any (genetically tractable) soft rot causing strains, particularly building upon pan-genomic analysis to identify key strains of interest.

In the strains tested here, many core metabolic processes were highly important in both minimal medium and tuber samples, such as biosynthesis of many amino acids. Notably, potato tubers contain higher arginine concentrations than other essential amino acids (Bártová et al., 2015), correlating with the dispensability of the arginine biosynthetic genes (*argCEFGH*) in tubers (Figure 4). Interestingly, though an *Erwinia amylovora argD* mutant is auxotrophic and non-pathogenic in apple (Ramos et al., 2014), *argD* is present in two apparently redundant copies in *Dda*3937 (DDA3937_RS19450 and DDA3937_RS03635), and mutants in these genes had no phenotype in the conditions tested. Previous studies have shown the important of chemotaxis and motility for early stage virulence (Jahn et al., 2008), but these traits were dispensable for growth in tubers with the inoculation and sampling methods used here. We used potato dextrose broth as an *in vitro* condition to mimic the nutritional composition of potato tubers more closely. This medium was slightly acidic, reflecting conditions encountered in the tuber or apoplast (Grignon and Sentenac, 1991; Kiszonas and Bamberg, 2010). Of the PDB-specific fitness factors, the low affinity potassium transporter Kup was likely required because of its role in ion transport particularly at low pH (Trchounian and Kobayashi, 1999).

Bacterial growth on potato tubers requires plant-specific virulence traits. Interestingly, the gene fitness profiles were indistinguishable among the potato cultivars tested. It is possible that tubers of alternative cultivars might sufficiently differ in their chemical composition to alter the metabolic genes needed for colonization. It would be useful to see if these results are consistent across additional plant tissues, such as the stem. Our data generally support *in planta* findings from other groups, from the importance of core metabolic capabilities during tuber colonization to the production of the pectin degradation protein KduD (Condemine and Robert-Baudouy, 1991; Royet et al., 2019; Czajkowski et al., 2020). Transposon mutagenesis of *P. carotovorum* followed by screening for altered soft rot symptoms in Chinese cabbage identified homologous genes involved in nutrient utilization, production of plant cell wall degrading enzymes, motility, biofilm formation, and toxin susceptibility (Lee et al., 2013). In chicory, *Dda*3937 required core metabolic and nucleic acid biosynthetic genes, flagellar motility, and iron uptake genes for full virulence (Royet et al., 2019). As in Royet et al. (2019) study, type III secretion system genes were dispensable for virulence when inoculated at high concentrations here. Type III effectors may promote initial multiplication within the host, but their role in *Dickeya* necrotrophic growth is secondary to the production of plant cell wall degrading enzymes (Yang et al., 2010). Though pectate lyases are collectively important for virulence (Condemine and Robert-Baudouy, 1991; Nachin and Barras, 2000), their redundancy makes mutagenesis-based study difficult (Schoedel and Collmer, 1986; Lojkowska et al., 1995). In all three *Dickeya* strains tested here, each of six pectate lyase genes were individually dispensable, supporting functional redundancy and/or complementation *in trans* (Supplementary Figure 6).

While *D. dadantii* and *D. dianthicola* can cause soft rot on potato tubers, variation in some key genes under these conditions suggests species- and strain-level differences in virulence strategies and stress responses. General strategies for environmental growth and host colonization are consistent, such as general metabolic capabilities and stress tolerance. However, gene fitness data suggest variation in gene regulation, such as the helix-turn-helix and other putative DNA-binding proteins. Large positive fitness values in *Dda*3937 flagellar mutants *in vitro* suggest that in liquid minimal medium flagella are costly. Since the same phenotype is not observed for *Ddia*ME23 or *Ddia*6719, this might imply flagella are controlled differently in liquid media in these species. Further characterization of the regulation of these traits is needed.

The scalability of RB-TnSeq, paired with ortholog identification, has proven to be a useful method to directly compare gene fitness between related strains. *Dickeya* species generally have common virulence strategies, primarily the production of plant cell wall degrading enzymes such as pectate lyases (Reverchon et al., 2016). However, genomic and transcriptomic variation at the strain and species level highlights distinctive virulence traits (Raoul des Essarts et al., 2019). This supports the intriguing possibility that while enzymatic virulence traits are shared across pathogens, there exists strain-specific virulence regulation. This idea has been proposed, but not directly tested, in *D. solani* based on predicted binding sites for transcriptional regulators (Golanowska et al., 2018). In the case of our study, while *Dda*3937 is pathogenic on potato, it was originally isolated from *Saintpaulia ionantha* (Lemattre and Narcy, 1972), suggesting potato infection is simply opportunistic. *Ddia*6719 was originally isolated from New Guinea impatiens (*Impatiens hawkeri*) (Liu et al., 2020a), but observed symptoms in tubers were similar to those caused by *Ddia*ME23.

This study focused on isolated strain growth, to generate a comprehensive dataset of likely essential genes and soft rot virulence traits in *D. dadantii* and *D. dianthicola*. Testing other *Dickeya* species, and closely related pathogens such as *Pectobacterium* spp., will more broadly expand our understanding of soft rot pathogens. With interest in using biocontrol strains or applying antimicrobial small molecules directly for control of soft rot (Czajkowski et al., 2011), it would be useful to screen pathogenic strains for potential resistance mechanisms. Similarly, in the field, soft rot symptoms can be the result of complex community interactions, with *Dickeya* and *Pectobacterium* co-infections frequently observed (Ge et al., 2021a). This presence of additional community members might change the composition of genes required for full competitive fitness. Understanding bacterial virulence strategies will aid in breeding efforts, as well as identify potential bacterial traits that could enable overcoming host tolerance or exacerbating disease at all stages of production. Similarly, the ability to rapidly predict and identify specific virulence traits in novel isolates will be key to addressing emerging outbreak pathogens.

## Data Availability Statement

All raw Illumina reads used for mapping and fitness assays have been deposited in the Sequence Read Archive under BioProject accession number PRJNA692477. Individual sample accession numbers are listed in Supplementary Table 2. Annotated scripts used for computational analysis are available at github.com/tylerhelmann/dickeya-barseq-2021. Experimental fitness values are publicly available at fit.genomics.lbl.gov.

## Author Contributions

TH, MF, and PS contributed to conception and designed of the study. TH performed the experiments and wrote the first draft of the manuscript. TH and PS performed data analysis. MF and PS supervised the project. All authors contributed to data interpretation, contributed to manuscript revision, read, and approved the submitted version.

## Conflict of Interest

The authors declare that the research was conducted in the absence of any commercial or financial relationships that could be construed as a potential conflict of interest.

## Publisher’s Note

All claims expressed in this article are solely those of the authors and do not necessarily represent those of their affiliated organizations, or those of the publisher, the editors and the reviewers. Any product that may be evaluated in this article, or claim that may be made by its manufacturer, is not guaranteed or endorsed by the publisher.

## References

[B1] Adeolu M. Alnajar S. Naushad S. Gupta R. S. (2016). Genome-based phylogeny and taxonomy of the ‘*Enterobacteriales*’: proposal for *Enterobacterales* ord. nov. divided into the families *Enterobacteriaceae*, *Erwiniaceae* fam. nov., *Pectobacteriaceae* fam. nov. *Int. J. Syst. Evol. Microbiol.* 66 5575–5599. 10.1099/ijsem.0.001485 27620848

[B2] Adriaenssens E. M. van Vaerenbergh J. Vandenheuvel D. Dunon V. Ceyssens P. J. de Proft M. (2012). T4-related bacteriophage LIMEstone isolates for the control of soft rot on potato caused by “Dickeya solani.”. *PLoS One* 7:e33227. 10.1371/journal.pone.0033227 22413005 PMC3296691

[B3] Bártová V. Bárta J. Brabcová A. Zdráhal Z. Horáčková V. (2015). Amino acid composition and nutritional value of four cultivated South American potato species. *J. Food Compos. Anal.* 40 78–85. 10.1016/j.jfca.2014.12.006

[B4] Bertani G. (1951). Studies on lysogenesis. I. The mode of phage liberation by lysogenic *Escherichia coli*. *J. Bacteriol.* 62 293–300. 10.1128/JB.62.3.293-300.1951 14888646 PMC386127

[B5] Chen I. M. A. Chu K. Palaniappan K. Pillay M. Ratner A. Huang J. (2019). IMG/M v.5.0: an integrated data management and comparative analysis system for microbial genomes and microbiomes. *Nucleic Acids Res.* 47 D666–D677. 10.1093/nar/gky901 30289528 PMC6323987

[B6] Chung Y. S. Goeser N. J. Cai X. Jansky S. (2013). The effect of long term storage on bacterial soft rot resistance in potato. *Am. J. Potato Res.* 90 351–356. 10.1007/s12230-013-9311-6

[B7] Cole B. J. Feltcher M. E. Waters R. J. Wetmore K. M. Mucyn T. S. Ryan E. M. (2017). Genome-wide identification of bacterial plant colonization genes. *PLoS Biol.* 15:e2002860. 10.1371/journal.pbio.2002860 28938018 PMC5627942

[B8] Condemine G. Robert-Baudouy J. (1991). Analysis of an *Erwinia chrysanthemi* gene cluster involved in pectin degradation. *Mol. Microbiol.* 5 2191–2202. 10.1111/j.1365-2958.1991.tb02149.x 1766386

[B9] Czajkowski R. Fikowicz-Krosko J. Maciag T. Rabalski L. Czaplewska P. Jafra S. (2020). Genome-wide identification of *Dickeya solani* transcriptional units up-regulated in response to plant tissues from a crop-host *Solanum tuberosum* and a weed-host *Solanum dulcamara*. *Front. Plant Sci.* 11:580330. 10.3389/fpls.2020.580330 32983224 PMC7492773

[B10] Czajkowski R. Pérombelon M. C. M. Van Veen J. A. Van der Wolf J. M. (2011). Control of blackleg and tuber soft rot of potato caused by *Pectobacterium* and *Dickeya* species: a review. *Plant Pathol.* 60 999–1013. 10.1111/j.1365-3059.2011.02470.x

[B11] Czajkowski R. Smolarska A. Ozymko Z. (2017). The viability of lytic bacteriophage ΦD5 in potato-associated environments and its effect on *Dickeya solani* in potato (*Solanum tuberosum* L.) plants. *PLoS One* 12:e183200. 10.1371/journal.pone.0183200 28800363 PMC5553641

[B12] Ge T. Jiang H. Tan E. H. Johnson S. B. Larkin R. Charkowski A. O. (2021c). Pangenomic analysis of *Dickeya dianthicola* strains related to the outbreak of blackleg and soft rot of potato in USA. *Plant Dis. [Online ahead of print]* DIS03210587RE. 10.1094/PDIS-03-21-0587-RE 34213964

[B13] Ge T. Jiang H. Johnson S. B. Larkin R. P. Charkowski A. O. Secor G. (2021b). Genotyping *Dickeya dianthicola* causing potato blackleg and soft rot outbreak associated with inoculum geography in the United States. *Plant Dis.* 105 1976–1983. 10.1094/PDIS-10-20-2138-RE 33210970

[B14] Ge T. Ekbataniamiri F. Johnson S. B. Larkin R. P. Hao J. (2021a). Interaction between *Dickeya dianthicola* and *Pectobacterium parmentieri* in potato infection under field conditions. *Microorganisms* 9 1–10. 10.3390/microorganisms9020316 33557052 PMC7913861

[B15] Georgoulis S. Shalvarjian K. E. Helmann T. C. Hamilton C. D. Carlson H. K. Deutschbauer A. M. (2021). Genome-wide identification of tomato xylem sap fitness factors for three plant-pathogenic *Ralstonia* species. *mSystems* 6 e1229–e1221. 10.1101/2020.08.31.276741 PMC8562481 34726495

[B16] Golanowska M. Potrykus M. Motyka-Pomagruk A. Kabza M. Bacci G. Galardini M. (2018). Comparison of highly and weakly virulent *Dickeya solani* strains, with a view on the pangenome and panregulon of this species. *Front. Microbiol.* 9:1940. 10.3389/fmicb.2018.01940 30233505 PMC6127512

[B17] Grignon C. Sentenac H. (1991). pH and ionic conditions in the apoplast. *Annu. Rev. Plant Physiol. Plant Mol. Biol.* 42 103–128. 10.1146/annurev.pp.42.060191.000535

[B18] Helmann T. C. Deutschbauer A. M. Lindow S. E. (2019). Genome-wide identification of *Pseudomonas syringae* genes required for fitness during colonization of the leaf surface and apoplast. *Proc. Natl. Acad. Sci. U.S.A.* 116 18900–18910. 10.1073/pnas.1908858116 31484768 PMC6754560

[B19] Jahn C. E. Willis D. K. Charkowski A. O. (2008). The flagellar sigma factor FliA is required for *Dickeya dadantii* virulence. *Mol. Plant-Microbe Interact.* 21 1431–1442. 10.1094/MPMI-21-11-1431 18842093

[B20] Jiang X. Zghidi-Abouzid O. Oger-Desfeux C. Hommais F. Greliche N. Muskhelishvili G. (2016). Global transcriptional response of *Dickeya dadantii* to environmental stimuli relevant to the plant infection. *Environ. Microbiol.* 18 3651–3672. 10.1111/1462-2920.13267 26940633

[B21] Kiszonas A. M. Bamberg J. B. (2010). Survey of tuber pH variation in potato (*Solanum*) species. *Am. J. Potato Res.* 87 167–176. 10.1007/s12230-009-9120-0

[B22] Lee D. H. Lim J. A. Lee J. Roh E. Jung K. Choi M. (2013). Characterization of genes required for the pathogenicity of *Pectobacterium carotovorum* subsp. *carotovorum* Pcc21 in Chinese cabbage. *Microbiology (United Kingdom)* 159 1487–1496. 10.1099/mic.0.067280-0 23676432 PMC3749726

[B23] Lemattre M. Narcy J. P. (1972). Une affection bacterienne nouvelle du Saintpaulia due a *Erwinia chrysanthemi*. *C. R. Acad. Sci* 58 227–231.

[B24] Liu H. Deutschbauer A. M. (2018). Rapidly moving new bacteria to model-organism status. *Curr. Opin. Biotechnol.* 51 116–122. 10.1016/j.copbio.2017.12.006 29316481

[B25] Liu H. Shiver A. L. Price M. N. Carlson H. K. Trotter V. V. Chen Y. (2021). Functional genetics of human gut commensal *Bacteroides thetaiotaomicron* reveals metabolic requirements for growth across environments. *Cell Rep.* 34:108789. 10.1016/j.celrep.2021.108789 33657378 PMC8121099

[B26] Liu Y. Helmann T. Stodghill P. Filiatrault M. (2020a). Complete genome sequence resource for the necrotrophic plant-pathogenic bacterium *Dickeya dianthicola* 67-19 isolated from New Guinea Impatiens. *Plant Dis.* 105 1174–1176. 10.1094/PDIS-09-20-1968-A 33064625

[B27] Liu Y. Vasiu S. Daughtrey M. L. Filiatrault M. (2020b). First Report of *Dickeya dianthicola* causing blackleg on New Guinea Impatiens (*Impatiens hawkeri*) in New York State, USA. *Plant Dis. [Online ahead of print]* 10.1094/pdis-09-20-2020-pdn 33200972

[B28] Lojkowska E. Masclaux C. Boccara M. Robert-Baudouy J. Hugouvieux-Cotte-Pattat N. (1995). Characterization of the *pelL* gene encoding a novel pectate lyase of *Erwinia chrysanthemi* 3937. *Mol. Microbiol.* 16 1183–1195. 8577252 10.1111/j.1365-2958.1995.tb02341.x

[B29] Lyon G. D. (1989). The biochemical basis of resistance of potatoes to soft rot *Erwinia* spp.—a review. *Plant Pathol.* 38 313–339. 10.1111/j.1365-3059.1989.tb02152.x

[B30] M9 Minimal Medium (Standard) (2010). M9 minimal medium (standard). *Cold Spring Harb. Protoc.* 2010:db.rec12295. 10.1101/pdb.rec12295

[B31] Ma X. Perna N. T. Glasner J. D. Hao J. Johnson S. Nasaruddin A. S. (2019). Complete genome sequence of *Dickeya dianthicola* ME23, a pathogen causing blackleg and soft rot diseases of potato. *Microbiol. Resour. Announc.* 8 14–15. 10.1128/mra.01526-18 30801063 PMC6376422

[B32] Melnyk R. A. Hossain S. S. Haney C. H. (2019). Convergent gain and loss of genomic islands drives lifestyle changes in plant-associated *Pseudomonas*. *ISME J.* 13 1575–1588. 10.1101/345488 30787396 PMC6776051

[B33] Motyka A. Zoledowska S. Sledz W. Lojkowska E. (2017). Molecular methods as tools to control plant diseases caused by *Dickeya* and *Pectobacterium* spp: a minireview. *N. Biotechnol.* 39 181–189. 10.1016/j.nbt.2017.08.010 28847714

[B34] Motyka-Pomagruk A. Zoledowska S. Misztak A. E. Sledz W. Mengoni A. Lojkowska E. (2020). Comparative genomics and pangenome-oriented studies reveal high homogeneity of the agronomically relevant enterobacterial plant pathogen *Dickeya solani*. *BMC Genomics* 21:449. 10.1186/s12864-020-06863-w 32600255 PMC7325237

[B35] Nachin L. Barras F. (2000). External pH: an environmental signal that helps to rationalize pel gene duplication in *Erwinia chrysanthemi*. *Mol. Plant-Microbe Interact.* 13 882–886. 10.1094/MPMI.2000.13.8.882 10939260

[B36] Parkinson N. DeVos P. Pirhonen M. Elphinstone J. (2014). *Dickeya aquatica* sp. nov., isolated from waterways. *Int. J. Syst. Evol. Microbiol.* 64 2264–2266. 10.1099/ijs.0.058693-0 24719023

[B37] Price M. N. Wetmore K. M. Waters R. J. Callaghan M. Ray J. Liu H. (2018). Mutant phenotypes for thousands of bacterial genes of unknown function. *Nature* 557 503–509. 10.1038/s41586-018-0124-0 29769716

[B38] R Core Team (2017). *R: A Language and Environment for Statistical Computing.* Vienna: R Foundation for Statistical Computing.

[B39] Ramos L. S. Lehman B. L. Peter K. A. McNellis T. W. (2014). Mutation of the *Erwinia amylovora argD* gene causes arginine auxotrophy, nonpathogenicity in apples, and reduced virulence in pears. *Appl. Environ. Microbiol.* 80 6739–6749. 10.1128/AEM.02404-14 25172854 PMC4249043

[B40] Raoul des Essarts Y. Pédron J. Blin P. Van Dijk E. Faure D. Van Gijsegem F. (2019). Common and distinctive adaptive traits expressed in *Dickeya dianthicola* and *Dickeya solani* pathogens when exploiting potato plant host. *Environ. Microbiol.* 21 1004–1018. 10.1111/1462-2920.14519 30618082

[B41] Reverchon S. Muskhelisvili G. Nasser W. (2016). Virulence program of a bacterial plant pathogen: the Dickeya model. *Progr. Mol. Biol. Transl. Sci.* 142 51–92. 10.1016/bs.pmbts.2016.05.005 27571692

[B42] Royet K. Parisot N. Rodrigue A. Gueguen E. Condemine G. (2019). Identification by Tn-seq of *Dickeya dadantii* genes required for survival in chicory plants. *Mol. Plant Pathol.* 20 287–306. 10.1111/mpp.12754 30267562 PMC6637903

[B43] Rubin B. E. Diamond S. Cress B. F. Crits-Christoph A. Lou Y. C. Borges A. L. (2021). Species- and site-specific genome editing in complex bacterial communities. *Nat. Microbiol.* 7 34–47. 10.1038/s41564-021-01014-7 34873292 PMC9261505

[B44] Samson R. Legendre J. B. Christen R. Fischer-Le Saux M. Achouak W. Gardan L. (2005). Transfer of *Pectobacterium chrysanthemi* (Burkholder et al. 1953) Brenner et al. 1973 and *Brenneria paradisiaca* to the genus *Dickeya* gen. nov. as *Dickeya chrysanthemi* comb. nov. and *Dickeya paradisiaca* comb. nov. *Int. J. Syst. Evol. Microbiol.* 55 1415–1427. 10.1099/ijs.0.02791-0 16014461

[B45] Schoedel C. Collmer A. (1986). Evidence of homology between the pectate lyase-encoding *pelB* and *pelC* genes in *Erwinia chrysanthemi*. *J. Bacteriol.* 167 117–123. 10.1128/jb.167.1.117-123.1986 3013832 PMC212849

[B46] Tian Y. Zhao Y. Yuan X. Yi J. Fan J. Xu Z. (2016). *Dickeya fangzhongdai* sp. nov., a plant-pathogenic bacterium isolated from pear trees (*Pyrus pyrifolia*). *Int. J. Syst. Evol. Microbiol.* 66 2831–2835. 10.1099/ijsem.0.001060 27045848

[B47] Toth I. K. van der Wolf J. M. Saddler G. Lojkowska E. Hélias V. Pirhonen M. (2011). *Dickeya* species: an emerging problem for potato production in Europe. *Plant Pathol.* 60 385–399. 10.1111/j.1365-3059.2011.02427.x

[B48] Trchounian A. Kobayashi H. (1999). Kup is the major K+ uptake system in *Escherichia coli* upon hyper-osmotic stress at a low pH. *FEBS Lett.* 447 144–148. 10.1016/S0014-5793(99)00288-4 10214935

[B49] van der Wolf J. M. Acuña I. De Boer S. H. Brurberg M. B. Cahill G. Charkowski A. O. (2021). “Diseases caused by *Pectobacterium* and *Dickeya* species around the world,” in *Plant Diseases Caused by Dickeya and Pectobacterium Species*, eds Van Gijsegem F. van der Wolf J. M. Toth I. K. (Cham: Springer International Publishing), 215–261.

[B50] Van Der Wolf J. M. Nijhuis E. H. Kowalewska M. J. Saddler G. S. Parkinson N. Elphinstone J. G. (2014). *Dickeya solani* sp. nov., a pectinolytic plant-pathogenic bacterium isolated from potato (*Solanum tuberosum*). *Int. J. Syst. Evol. Microbiol.* 64 768–774. 10.1099/ijs.0.052944-0 24225027

[B51] Van Gijsegem F. Toth I. K. van der Wolf J. M. (2021). “Outlook-Challenges and perspectives for management of diseases caused by *Pectobacterium* and *Dickeya* species,” in *Plant Diseases Caused by Dickeya and Pectobacterium Species*, eds Van Gijsegem F. van der Wolf J. M. Toth I. K. (Cham: Springer International Publishing), 283–289.

[B52] van Opijnen T. Bodi K. L. Camilli A. (2009). Tn-seq: high-throughput parallel sequencing for fitness and genetic interaction studies in microorganisms. *Nat. Methods* 6 767–772. 10.1038/nmeth.1377 19767758 PMC2957483

[B53] Wetmore K. M. Price M. N. Waters R. J. Lamson J. S. He J. Hoover C. A. (2015). Rapid quantification of mutant fitness in diverse bacteria by sequencing randomly bar-coded transposons. *MBio* 6 1–15. 10.1128/mBio.00306-15 25968644 PMC4436071

[B54] Wickham H. (2016). *ggplot2: Elegant Graphics for Data Analysis.* New York, NY: Springer-Verlag.

[B55] Yang S. Peng Q. Zhang Q. Zou L. Li Y. Robert C. (2010). Genome-wide identification of HrpL-regulated genes in the necrotrophic phytopathogen *Dickeya dadantii* 3937. *PLoS One* 5:e13472. 10.1371/journal.pone.0013472 20976052 PMC2957411

[B56] Zhang Y. Fan Q. Loria R. (2016). A re-evaluation of the taxonomy of phytopathogenic genera *Dickeya* and *Pectobacterium* using whole-genome sequencing data. *Syst. Appl. Microbiol.* 39 252–259. 10.1016/j.syapm.2016.04.001 27130313

