# Supplementary material for: Genome-Wide Identification of Genes Important for Growth of Dickeya dadantii and Dickeya dianthicola in Potato (Solanum tuberosum) Tubers
Source: Front Microbiol. 2022 Jan 25;13:778927. doi: 10.3389/fmicb.2022.778927 (PMC8821946; doi:10.3389/fmicb.2022.778927)

Supplementary Figure 1. Distribution of mapped strains for barcoded *mariner* transposon insertion mutant libraries in *D. dadantii* 3937 (Ddi3937), *D. dianthicola* ME23 (DdiaME23), and *D. dianthicola* 67-19 (Ddia6719).

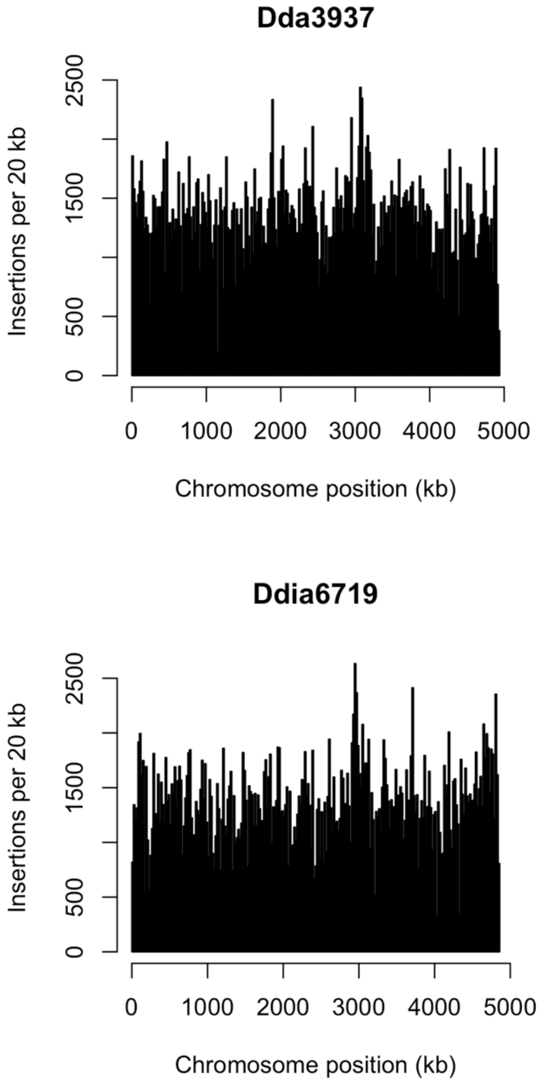

Supplement: Supplementary file 1 [file Image_1.PDF]
