# Supplementary material for: Genome-Wide Identification of Genes Important for Growth of Dickeya dadantii and Dickeya dianthicola in Potato (Solanum tuberosum) Tubers
Source: Front Microbiol. 2022 Jan 25;13:778927. doi: 10.3389/fmicb.2022.778927 (PMC8821946; doi:10.3389/fmicb.2022.778927)

Supplementary Figure 2. Most predicted essential genes are common among all three strains. Overlap was calculated based on PyParanoid group numbers for each predicted essential gene. Due to the insertion density of each library, to predict a gene as essential size cutoffs of >175 bp (*Dda*3937) and >150 bp (*Ddia*ME23 and *Ddia*6719) were used. Genes with no ortholog group assignments are not included here: N= 11 (*Dda*3937), 16 (*Ddia*ME23), and 19 (*Ddia*6719).

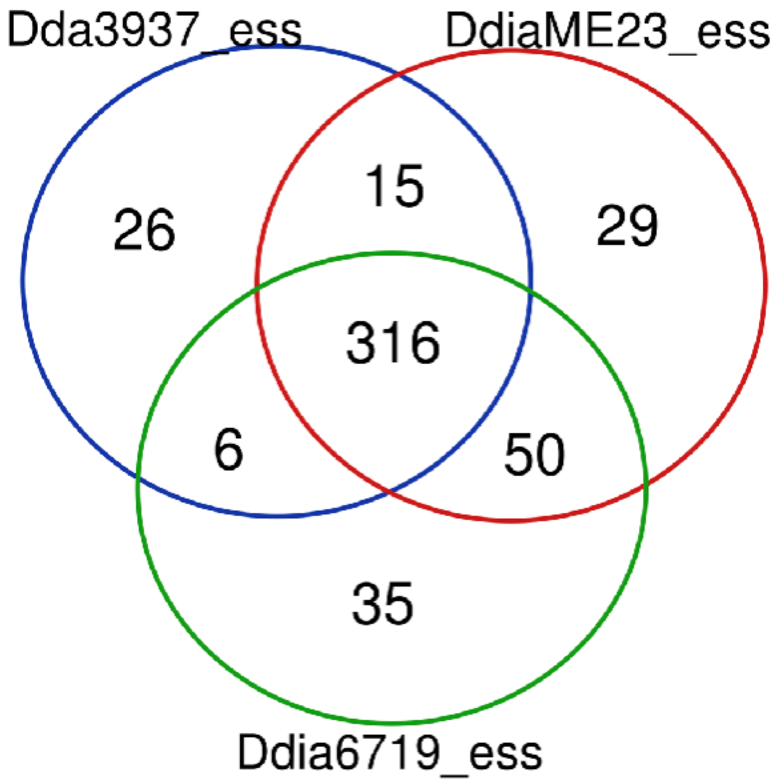

Supplement: Supplementary file 2 [file Image_2.PDF]
