# Supplementary material for: Genome-Wide Identification of Genes Important for Growth of Dickeya dadantii and Dickeya dianthicola in Potato (Solanum tuberosum) Tubers
Source: Front Microbiol. 2022 Jan 25;13:778927. doi: 10.3389/fmicb.2022.778927 (PMC8821946; doi:10.3389/fmicb.2022.778927)

Supplementary Figure 3. *In vitro* growth of wild-type *D. dadantii* 3937, *D. dianthicola* ME23, and *D. dianthicola* 67-19 in LB, Potato Dextrose Broth (PDB), and M9 minimal medium containing 0.4% glycerol. (**A**) Growth curve measure absorbance at 600 nm of each strain showing 6 replicate samples per media type. (**B**) Doubling time summary statistics calculated using the R package growthcurver v0.3.1 (Sprouffske and Wagner 2016).

A.

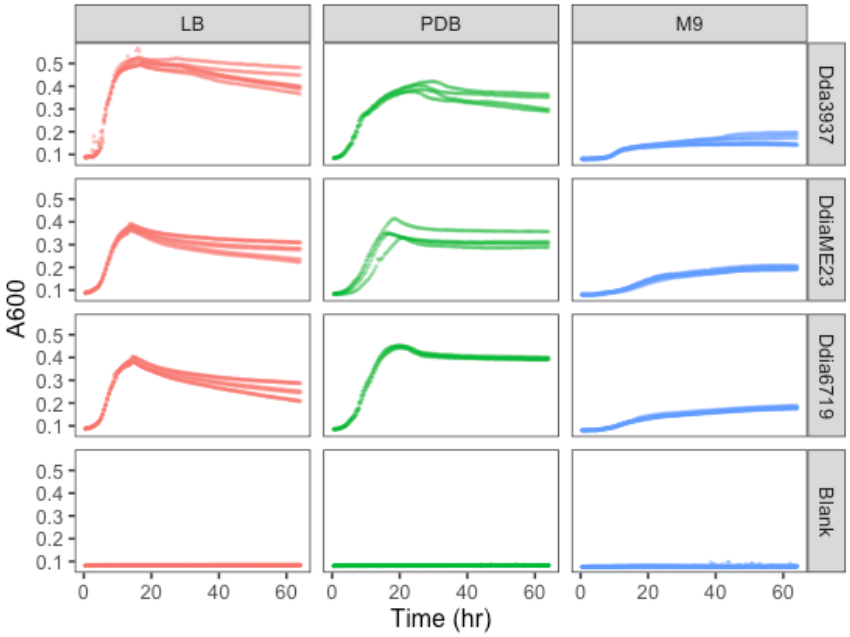

B.

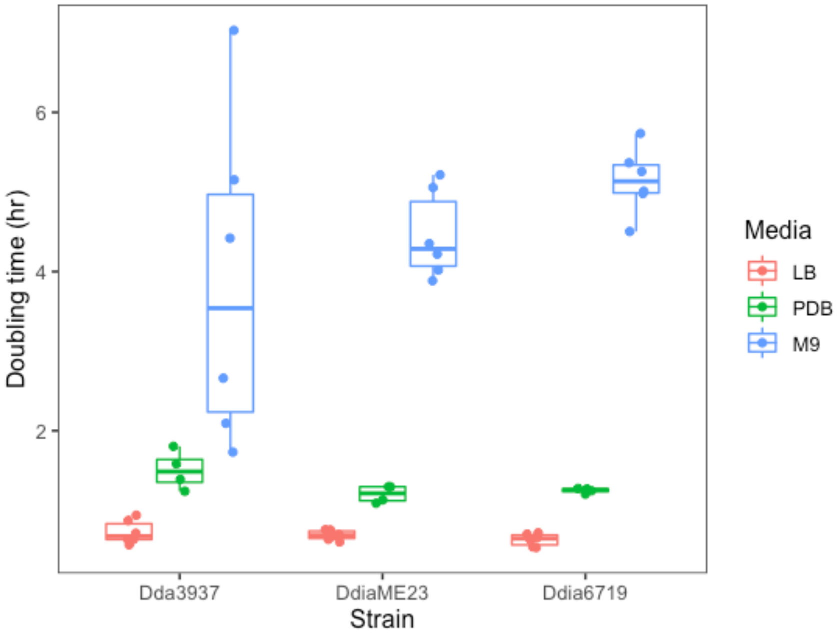

Supplement: Supplementary file 3 [file Image_3.PDF]
