# Supplementary material for: Genome-Wide Identification of Genes Important for Growth of Dickeya dadantii and Dickeya dianthicola in Potato (Solanum tuberosum) Tubers
Source: Front Microbiol. 2022 Jan 25;13:778927. doi: 10.3389/fmicb.2022.778927 (PMC8821946; doi:10.3389/fmicb.2022.778927)

Supplementary Figure 4. Gene fitness values for glucans biosynthesis proteins MdoGH (groups 00352 and 00097), cell division protein FtsX (group 01645), and cell division protein ZapB (group 03211).

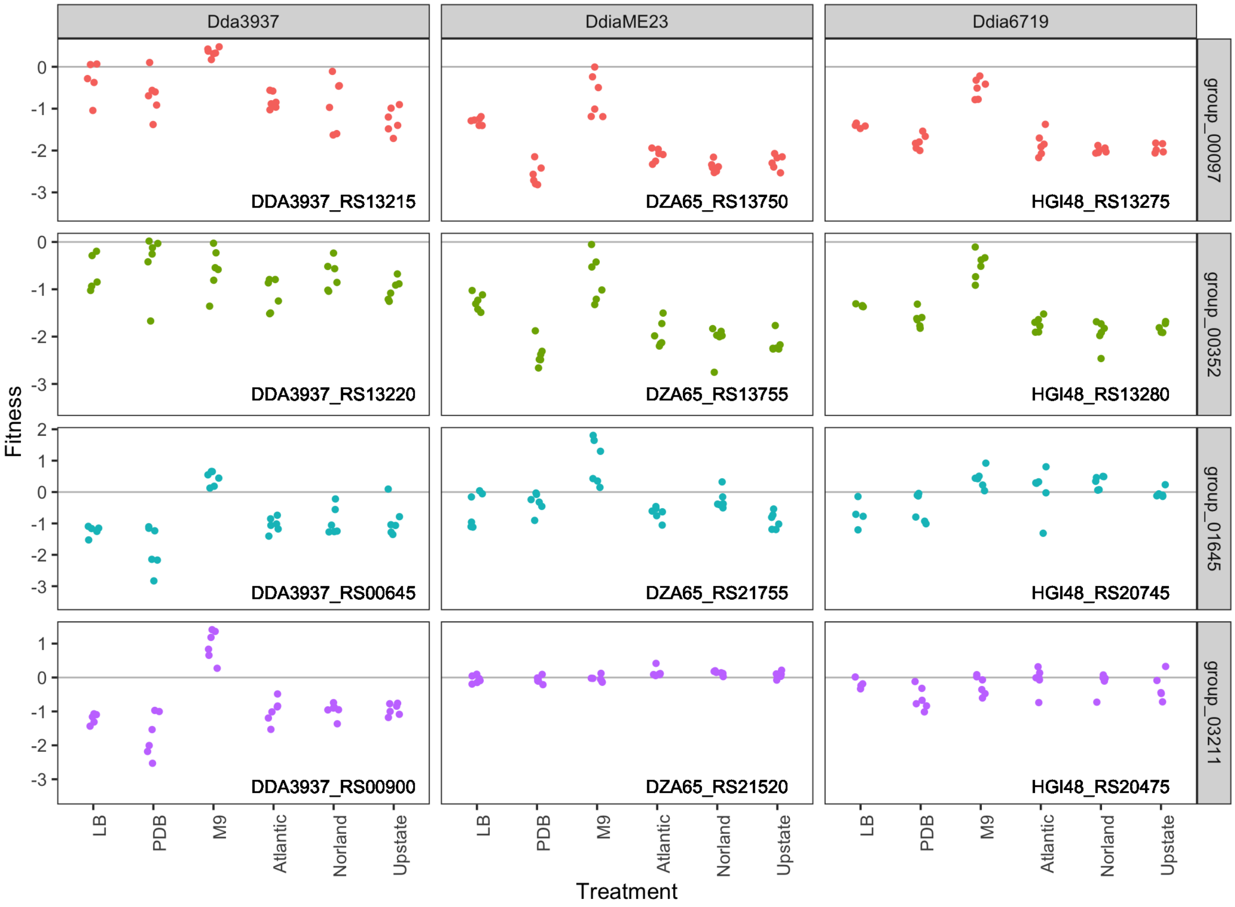

Supplement: Supplementary file 4 [file Image_4.PDF]
