# Supplementary material for: Genome-Wide Identification of Genes Important for Growth of Dickeya dadantii and Dickeya dianthicola in Potato (Solanum tuberosum) Tubers
Source: Front Microbiol. 2022 Jan 25;13:778927. doi: 10.3389/fmicb.2022.778927 (PMC8821946; doi:10.3389/fmicb.2022.778927)

Supplementary Figure 5. Gene fitness values for oligopeptidase A (group 00188), the low affinity potassium transporter Kup (group 00240), the two-component system RtsAB (groups 02048 and 00671), and the zinc uptake transcriptional repressor Zur (group 02565).

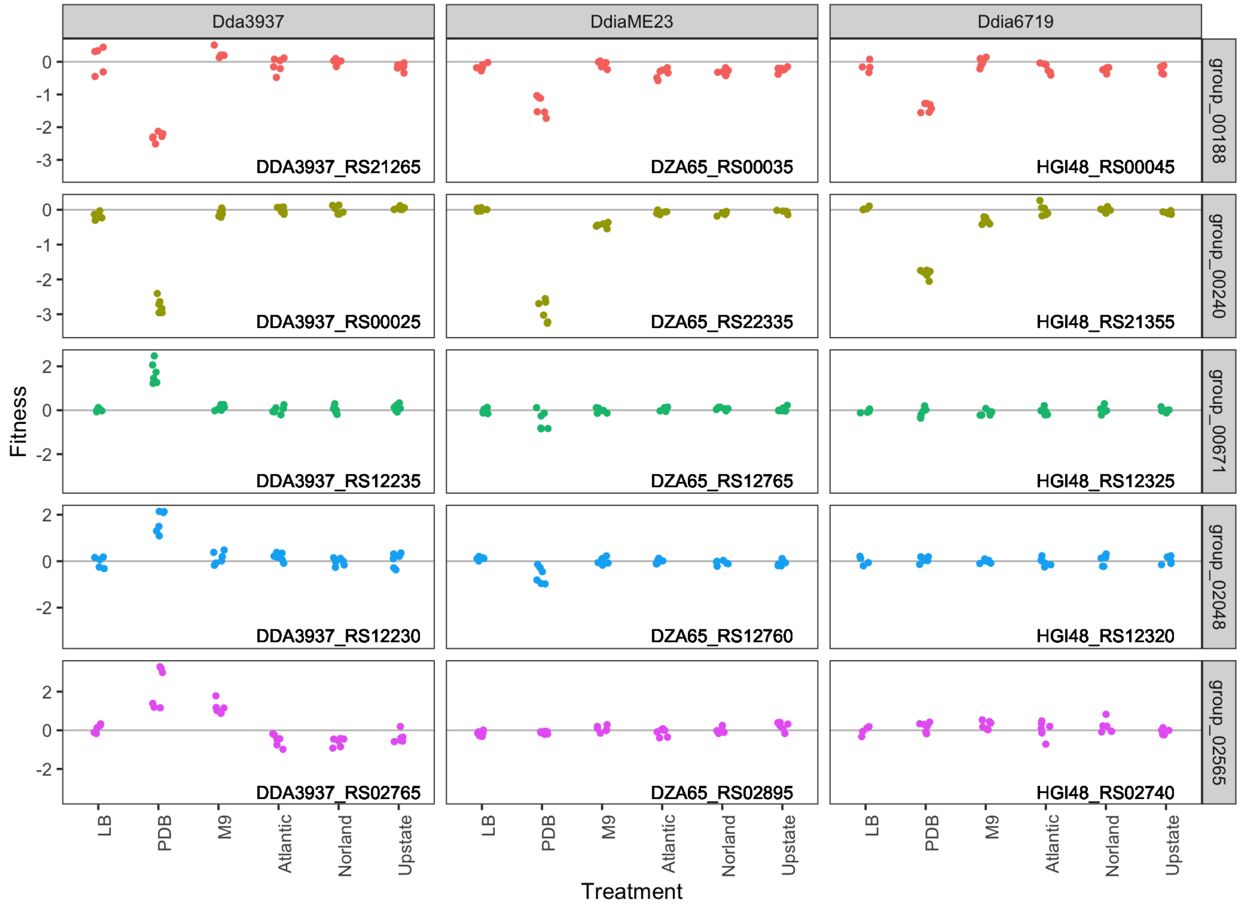

Supplement: Supplementary file 5 [file Image_5.PDF]
