# Supplementary material for: Genome-Wide Identification of Genes Important for Growth of Dickeya dadantii and Dickeya dianthicola in Potato (Solanum tuberosum) Tubers
Source: Front Microbiol. 2022 Jan 25;13:778927. doi: 10.3389/fmicb.2022.778927 (PMC8821946; doi:10.3389/fmicb.2022.778927)

Supplementary Figure 6. Individual pectate lyases are dispensable during tuber colonization. Gene fitness values for genes annotated as “pectate lyase” (groups 00306, 00799, 01227, 01397, 01488, and 01593). In *Ddia*6719, HGI48_RS13640 (group 00799) is annotated only as a “right-handed parallel beta-helix repeat-containing protein”.

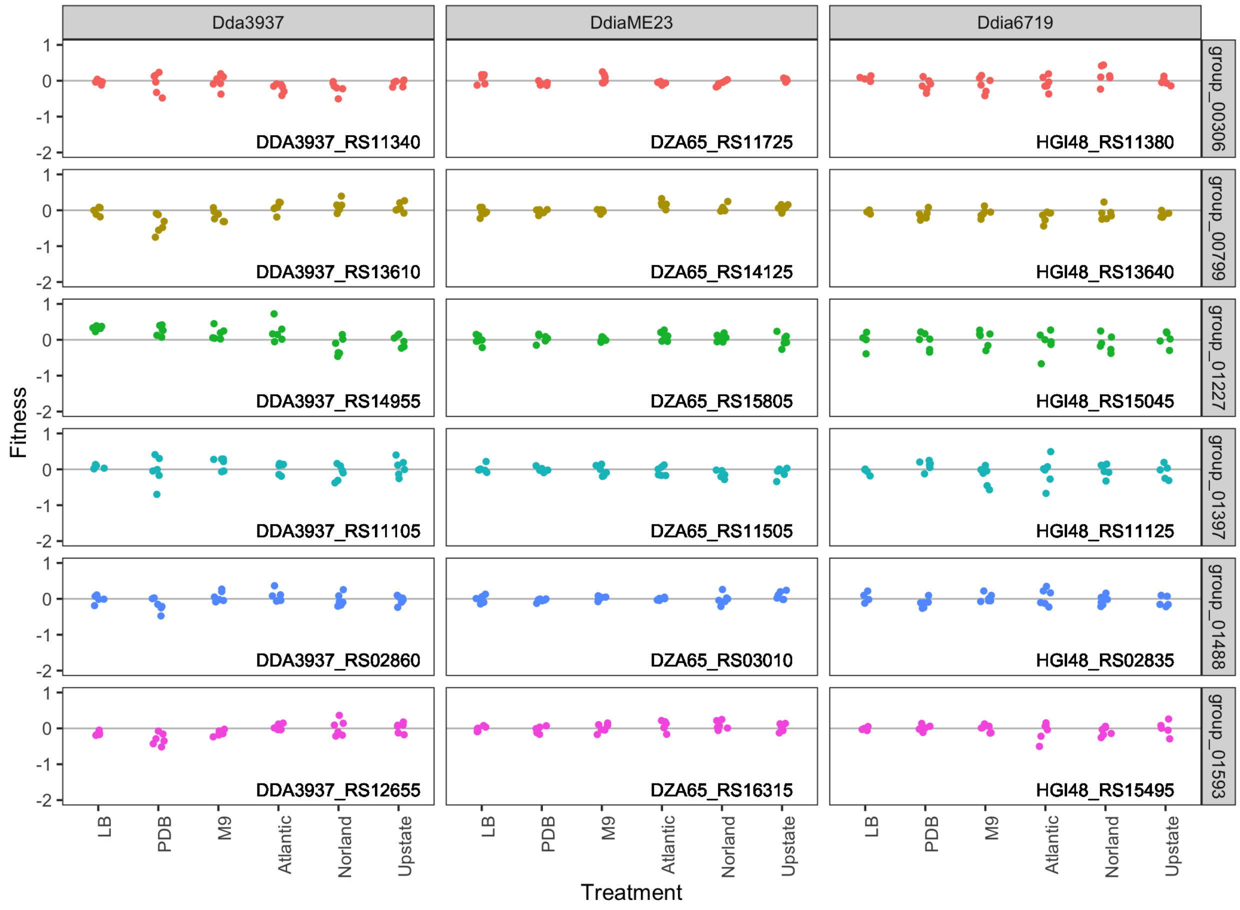

Supplement: Supplementary file 6 [file Image_6.PDF]
