# Supplementary material for: Genome-Wide Identification of Genes Important for Growth of Dickeya dadantii and Dickeya dianthicola in Potato (Solanum tuberosum) Tubers
Source: Front Microbiol. 2022 Jan 25;13:778927. doi: 10.3389/fmicb.2022.778927 (PMC8821946; doi:10.3389/fmicb.2022.778927)
Supplement: Supplementary file 7 [file Table_1.DOCX]

**Supplementary Table 1.** Strains used in this study.

| Strain | Description | Source |
| --- | --- | --- |
| *D. dadantii* 3937 | Wild-type strain | (Lemattre and Narcy, 1972; Samson et al., 2005) |
| *D. dianthicola* ME23 | Wild-type strain | (Ma et al., 2019) |
| *D. dianthicola* 67-19 | Wild-type strain | (Liu et al., 2020) |
| *E. coli* WM3064 | Strain APA752; barcoded *mariner* transposon vector (Kan^R^) in *E. coli* conjugation strain | (Wetmore et al., 2015) |
| *D. dadantii* 3937 | Whole genome barcoded *mariner* transposon library (KanR) | This work |
| *D. dianthicola* ME23 | Whole genome barcoded *mariner* transposon library (KanR) | This work |
| *D. dianthicola* 67-19 | Whole genome barcoded *mariner* transposon library (KanR) | This work |

Lemattre, M., and Narcy, J. P. (1972). Une affection bacterienne nouvelle du Saintpaulia due a *Erwinia chrysanthemi*. *C. R. Acad. Sci* 58, 227–231.

Liu, Y., Helmann, T., Stodghill, P., and Filiatrault, M. (2020). Complete genome sequence resource for the necrotrophic plant-pathogenic bacterium *Dickeya dianthicola* 67-19 isolated from New Guinea Impatiens. *Plant Dis.*, PDIS-09-20-1968-A. doi:10.1094/PDIS-09-20-1968-A.

Ma, X., Perna, N. T., Glasner, J. D., Hao, J., Johnson, S., Nasaruddin, A. S., et al. (2019). Complete genome sequence of *Dickeya dianthicola* ME23, a pathogen causing blackleg and soft rot diseases of potato. *Microbiol. Resour. Announc.* 8, 14–15. doi:10.1128/mra.01526-18.

Samson, R., Legendre, J. B., Christen, R., Fischer-Le Saux, M., Achouak, W., and Gardan, L. (2005). Transfer of *Pectobacterium chrysanthemi* (Burkholder et al. 1953) Brenner et al. 1973 and *Brenneria paradisiaca* to the genus *Dickeya* gen. nov. as *Dickeya chrysanthemi* comb. nov. and *Dickeya paradisiaca* comb. nov. ... *Int. J. Syst. Evol. Microbiol.* 55, 1415–1427. doi:10.1099/ijs.0.02791-0.

Wetmore, K. M., Price, M. N., Waters, R. J., Lamson, J. S., He, J., Hoover, C. A., et al. (2015). Rapid quantification of mutant fitness in diverse bacteria by sequencing randomly bar-coded transposons. *MBio* 6, 1–15. doi:10.1128/mBio.00306-15.
