# Supplementary material for: Genome-Wide Identification of Genes Important for Growth of Dickeya dadantii and Dickeya dianthicola in Potato (Solanum tuberosum) Tubers
Source: Front Microbiol. 2022 Jan 25;13:778927. doi: 10.3389/fmicb.2022.778927 (PMC8821946; doi:10.3389/fmicb.2022.778927)
Supplement: Supplementary file 11 [file Table_5.DOCX]

**Supplementary Table 5.** Number of genes within each functional category predicted to be essential or nearly essential in LB, based on the TnSeq data. Protein coding genes were considered essential or nearly essential for growth in LB if normalized read density (reads/nucleotides across the entire gene) and normalized insertion density (sites/nucleotides in the central 10 to 90% of the gene) were below 0.2 (Price et al., 2018). Genes shorter than 175 bp (*Dda*3937) or 150 bp (*Ddia*ME23 and *Ddia*6719) were excluded from this analysis. Functional category annotations are COG assignments for *Dda*3937 genes in the IMG database (Chen et al., 2019), matched to PyParanoid-determined *Ddia*ME23 and *Ddia*6719 orthologs.

| **COG** | ***Dda*3937** | ***Ddia*ME23** | ***Ddia*6719** |
| --- | --- | --- | --- |
| Translation, ribosomal structure, and biogenesis | 87 | 102 | 102 |
| Cell wall/membrane/envelope biogenesis | 54 | 53 | 51 |
| Coenzyme transport and metabolism | 27 | 37 | 35 |
| Energy production and conversions | 26 | 26 | 24 |
| Replication, recombination, and repair | 25 | 29 | 30 |
| Lipid transport and metabolism | 23 | 23 | 23 |
| Cell cycle control, cell division, chromosome partitioning | 15 | 13 | 14 |
| Nucleotide transport and metabolism | 15 | 13 | 14 |
| None | 14 | 15 | 19 |
| Transcription | 14 | 15 | 15 |
| Intracellular trafficking, secretion, and vesicular transport | 12 | 9 | 10 |
| Posttranslational modification, protein turnover, chaperones | 11 | 15 | 13 |
| Amino acid transport and metabolism | 10 | 18 | 15 |
| Carbohydrate transport and metabolism | 10 | 13 | 12 |
| Inorganic ion transport and metabolism | 6 | 8 | 6 |
| General function prediction only | 4 | 2 | 3 |
| Secondary metabolites biosynthesis, transport, and catabolism | 3 | 2 | 2 |
| Signal transduction mechanisms | 3 | 5 | 5 |
| Function unknown | 2 | 2 | 5 |
| Defense mechanisms | 1 | 1 | 1 |
| RNA processing and modification | 1 | 1 | 1 |
| Cell motility | 0 | 0 | 0 |
| Extracellular structures | 0 | 0 | 0 |
| Mobilome: prophages, transposons | 0 | 0 | 0 |
| (No COG and/or orthogroup assignment) | 11 | 24 | 26 |
| **Total** | **374** | **426** | **426** |

Chen, I. M. A., Chu, K., Palaniappan, K., Pillay, M., Ratner, A., Huang, J., et al. (2019). IMG/M v.5.0: an integrated data management and comparative analysis system for microbial genomes and microbiomes. *Nucleic Acids Res.* 47, D666–D677. doi:10.1093/nar/gky901.

Price, M. N., Wetmore, K. M., Waters, R. J., Callaghan, M., Ray, J., Liu, H., et al. (2018). Mutant phenotypes for thousands of bacterial genes of unknown function. *Nature* 557, 503–509. doi:10.1038/s41586-018-0124-0.
